# Mental simulation of the factual and the illusory in negation processing: evidence from anticipatory eye movements on a blank screen

**DOI:** 10.1038/s41598-024-53353-0

**Published:** 2024-02-03

**Authors:** Norbert Vanek, Ana Matić Škorić, Sara Košutar, Štěpán Matějka, Kate Stone

**Affiliations:** 1 Charles University Prague, Prague, Czechia; 2 University of Auckland, Auckland, New Zealand; 3 University of Zagreb, Zagreb, Croatia; 4 UiT The Arctic University of Norway, Tromsø, Norway; 5 University of Potsdam, Potsdam, Germany

**Keywords:** Neuroscience, Psychology

## Abstract

How do comprehenders process negative statements such as *The fish is not jumping out of the water*? Opinions vary. Some argue for two steps, namely that processing starts off with the representation of the positive/illusory [fish jumping out of the water] and then shifts to the (f)actual. To test this idea, we measured fixations on the factual (fish not jumping) versus the illusory (fish jumping) during auditory processing of negation and affirmation. We tested speakers of English (single-cued negation) and Croatian (double-cued negation) and focused on anticipatory fixations in the absence of pictures to indicate the strength of mental simulations. Our findings contribute to negation processing research in two ways. First, dominant anticipatory fixations on the factual suggest a direct rather than a two-step process. Second, time-sensitive insights from two languages call for a finer-grained account of negation processing with negation-specific support of inferences of the factual over the illusory.

## Introduction

Imagine a fish *not* jumping out of water. Is the negative state of affairs more difficult to imagine than the opposite? The phenomenon when negative sentences take more time to process than their positive counterparts is known as the *negation effect*. Much of current research on how negation is understood converges in two points. The first common finding is the negation effect, often exhibited as slowdowns in response to negative compared to affirmative statements, and the second is that different forms of negation come with variation in processing costs^1–7^. However, there are some less agreed-upon aspects, such as whether comprehenders mentally represent the negative state directly (e.g., picturing a submerged fish when reading or hearing *The fish is not jumping out of the water*) or whether negation processing requires an additional step^4,5^ through the corresponding positive state (an airborne fish). The extra step in which individuals first need to simulate the positive fits with the embodied mental simulation view^8–10^, which proposes that complete grounding in sensorimotor experience is necessary to mentally recreate a given state of affairs. In this study, we rely on anticipatory eye movements to examine whether something that is not gets mentally simulated.

Research using anticipatory eye movements builds on the assumption that skillful listeners use accruing linguistic cues to generate predictions about probable dependencies with information that is likely to come next^11^. Probabilistic computations are not limited to verbal knowledge, but they also include non-linguistic (e.g., picture-based) information, forming the basis of what is known as the visual world paradigm^12,13^. For instance, when hearing the sentence, ‘*The man has drunk all*…’, more fixations gravitate towards a picture of an empty glass than a full glass. This study adopts the visual world paradigm to monitor looks towards pictures showing the factual or the illusory state of affairs. Rather than co-presenting the sentence and the visual scene, the time-course of anticipatory fixations were measured after participants had previewed the pictures, while they were listening to linguistic input, before the pictures reappeared. This approach, known as *the blank screen paradigm*^14^, has a twofold benefit for negation processing research. The presence of anticipatory eye movements in the absence of a visual scene can signal whether listeners mentally simulate situations when they process negation. And if they do, the trajectories of such eye movements can speak to the resonant issue of whether listeners first represent the positive/illusory or if they process the negative/factual state of affairs directly.

The one-step or two-step debate is additionally complexified by factors that are known to cause processing differences, such as language-specific structural cues in negation^15–18^. One such linguistic cue is *negative concord*^6,19–21^ a type of negation that languages like English and Croatian tolerate differently. Negative concord is a linguistic feature where a sentence contains multiple negative elements, but they contribute to just one semantic negation^22,23^. An example of negative concord in Croatian is ***Nitko ne sluša***, lit. ‘**Nobody** is **not** listening’ which translates as ‘**Nobody** is listening’, where two negative elements are used, but they semantically convey a single negative meaning. This study examines the consequences of processing negation of two types, one that structurally overlaps in Croatian and English (i.e., sentential negation with a single negative cue in both Croatian and English, as in *Dijete ne sluša* ‘The child **isn’t** listening’) and one where negation structures crosslinguistically vary (i.e., negative concord with two negative cues in Croatian, as in ***Nitko ne sluša,*** where both *nitko* ‘nobody’ and *ne* ‘not’ contribute to emphasise the negation, vs. the corresponding negative quantifier negation with a single negative cue in English, as in ***Nobody is listening***). While negative concord in Croatian is obligatory^24^, standard English does not permit it. Languages that do not permit negative concord are known as *double negation* languages^22^. In these languages, sentences that contain two or more negative elements effectively cancel each other out, leading to an affirmative meaning (as in *She doesn’t want no help* which creates a positive meaning ‘She wants some help’). However, recent research showed that contextual cues can bias acceptability and comprehensibility of English negated sentences towards a negative concord reading or double negation reading^25^. Still, the default interpretation of two negative elements in an English sentence presented without context is that of double negation. To date, no studies have compared the processing consequences of strict negative concord in Croatian and its equivalent in standard English. The present study integrates this crosslinguistic comparison to inform the theoretical debate about (the number of steps in) processing negation.

### Theories of negation processing

The temporal dynamic that underlies negation processing is a vigorously debated issue. There are two major accounts, the *two-step* and the *fusion/one-step* model. In the two-step model, negation is processed sequentially. Listeners begin with the mental representation of the positive/illusory, and then shift to the representation of the negative/factual. Support for this indirect route was reported in studies that observed some form of increased processing demands linked to the factual compared to the illusory^5,26–30^. In the fusion or one-step model, the representation of the negative/factual is automatic, processed directly, without having to represent the positive/illusory state of affairs first^31–34^.

Representation of negation substantially differs within the two models, and the difference lies in the need to represent the illusory, i.e., the positive state of affairs. This difference in mental representations can be aptly captured as a contrast between *iconic* vs. *symbolic*^33^. The two-step model aligns with iconicity, and more broadly with the embodiment theory, in the sense that negation processing only involves mental representations that are fully grounded in the listener’s sensorimotor experience. The first step in the mental simulation of, for instance ‘*The coconut is not broken*’, is the positive/illusory (a broken coconut), and only then proceeds the simulation of the negative/factual (a whole coconut). Sequential processing of both steps is required, even when the positive/illusory alternative may not be readily available, as in ‘*The fish is not jumping out of the water’*. For cases with unavailable opposites, the first step in the simulation sequence may be empty^8^ or unspecified^5^ (the fish could be swimming, spawning etc.). If listeners mentally simulate the illusory in the processing stream first, then the resulting mental representation of negation is indirect, hence iconic. If, however, the listeners represent only the negative/factual state of affairs, then the representation is in some way symbolic, for instance, via cross as a symbolic marker of falsity^35^. A test able to track the time course of mental simulations while negation is processed may be in an optimal position to arbitrate between the two accounts.

### The present study

This study builds on the assumption that linguistic structure can facilitate or hinder negation processing and thereby affect mental simulations amongst comprehenders. We addressed this claim by manipulating the factual and the illusory in negation to directly explore native speakers’ mental simulations. Our intention was to observe whether differences in sentential negation led to differences in negation processing between languages, Croatian and English, as well as between negation types within languages. For this purpose, we designed an eye-tracking experiment, using a combination of pictures and audio recordings. We manipulated Negation type and Language, and we measured *anticipatory fixations* (fixations to indicate early mental simulation of the factual vs. illusory and proportions of looks during auditory processing in the absence of pictures to indicate the strength of mental simulations) and *integratory fixations* (proportions of looks after reappearance of pictures). Unlike earlier negation processing studies, this study uses the degree of language-mediation of anticipatory eye movements using the blank screen paradigm^14^ in its quest to establish whether the eyes track the location of the factual from early on, or if they detour through the illusory, when negative sentences are processed.

We tested two sets of predictions, one within and one between languages. Within languages, our main hypothesis was that participants would tend to launch anticipatory eye-fixations towards the factual directly without fixating on the illusory first^31–34^. Following the logic that more structural cues within a sentence can lead to more robust anticipatory eye movements to targets^36,37^, within Croatian we expected increased and earlier fixations on the factual in negative concord, followed by sentential negation. And crosslinguistically, based on the idea that predictions are generated incrementally drawing on multiple sources at the earliest possible opportunity^12^, we expected a smaller processing difference for the two negation types in English (single cue in sentential negation vs single cue in negative quantifier negation) than in Croatian (single cue in sentential negation vs double cue in negative concord). Language of input is not a factor that we could manipulate within participants, so to maximise crosslinguistic comparability we analysed between-condition differences within languages and then only compared pattern similarity in those differences across languages. The mechanism we tested was whether negative concord provides an additional cue making the factual relatively more salient, thus reducing the illusory effect earlier than the other types of negation included in this study. We find this approach informative on two levels, not only for its potential to disentangle early/anticipatory from late/integratory comprehension processes in response to the one/two-step debate, but also for the time-sensitive insights it offers to test the relative impact that various negation types across and within languages have on supporting inferences of the factual over the illusory.

## Experiment 1: Native Croatian listeners

### Method

#### Participants

Forty-two native Croatian speakers (mean age = 22.5 years, range = 18.1–25.7 years, 36 females) took part in the experiment. They were students at the University of Zagreb, and they received a gift voucher for participation. All participants reported Croatian to be their dominant language, normal or corrected-to-normal vision, and no neurological and/or language impairments. An a priori power analysis was conducted using G*Power version 3.1.9.7^38^ to determine the minimum sample size required to test the main hypothesis that anticipatory fixations on the factual would significantly outweigh anticipatory fixations on the illusory, irrespective of negation condition. To reach a power of 0.8 for detecting a medium effect size estimate of Cohen’s *d* = 0.5 at a significance criterion *α* = 0.05 for two dependent means, the suggested adequate sample size was *N* = 34. We increased this number to 42, taking into consideration that not all participants initiate language-mediated anticipatory fixations on a blank screen during every trial^14^.

#### Materials

The materials were black-and-white pictures presented in pairs, followed by short audio-recorded sentences. The picture pairs were black-and-white drawings, one picture representing the factual state (e.g., whole coconut) and the other showing the illusory state (e.g., broken coconut) with respect to the information presented in the corresponding audio-recorded sentence (e.g., *Nobody broke the coconut*). 50% of the pictures originated from a normed database for psycholinguistic studies^39^. The other 50% were drawn as the factual or the illusory pairs of the normed pictures. The correct answer was always the factual picture. The size of each picture was 300 × 300 pixels.

The audios were recordings of different negation types (sentential vs. negative concord in Croatian; 20 sentences/type) with corresponding affirmation used as a control condition. In sum, 20 picture pairs were combined with three different sentence types, namely sentential negation (e.g., *Majmun nije razbio kokos* ‘The monkey didn’t break the coconut’), negative concord (e.g., *Nitko nije razbio kokos* ‘Nobody broke the coconut’, or literally ‘*Nobody didn’t break the coconut’) and affirmation (e.g., *Majmun je razbio kokos* ‘The monkey broke the coconut’). Twenty fillers were randomly mixed in with the targets. The fillers also contained two related pictures but did not involve negation in the audio (e.g*., Susjed je probao ispeći ribu i riba je izgorjela.* ‘The neighbour tried to grill the fish and the fish got burnt’). Each participant also saw four practice trials to become familiar with the task and setup. ‘Trial’ equals a single instance when a picture pair was co-presented with a corresponding audio sentence. There were 84 trials in total.

Two levels of randomisation and counterbalancing were employed. Trial order was pseudo-randomised across participants. Two sentences of the same sentence triplet (e.g., 'The monkey broke the coconut', 'Nobody broke the coconut') could not immediately follow each other. The position of pictures showing the factual and the illusory situation was counterbalanced. 50% of the participants saw 2/3 of the illusory pictures (within the triplet) on the left, and 1/3 on the right, while the other 50% of the participants saw 1/3 of the illusory pictures on the left and 2/3 on the right.

#### Procedure

First, informed consent was obtained from all participants in the study (approved by the University of Auckland Human Participants Ethics Committee, Ref. UAHPEC23370). The experimental procedure started with the following instructions: “You are going to see two pictures and hear a sentence. Pay careful attention to both. First, two pictures will appear side by side. Second, the pictures will disappear, and you will hear a sentence. After the end of the sentence, the pictures will reappear. Your task is to choose the picture that best corresponds to the sentence. Press the left arrow key if you choose the picture on the left, or the right arrow key if you choose the picture on the right. Decide as fast and as accurately as you can.”

**Figure 1:**
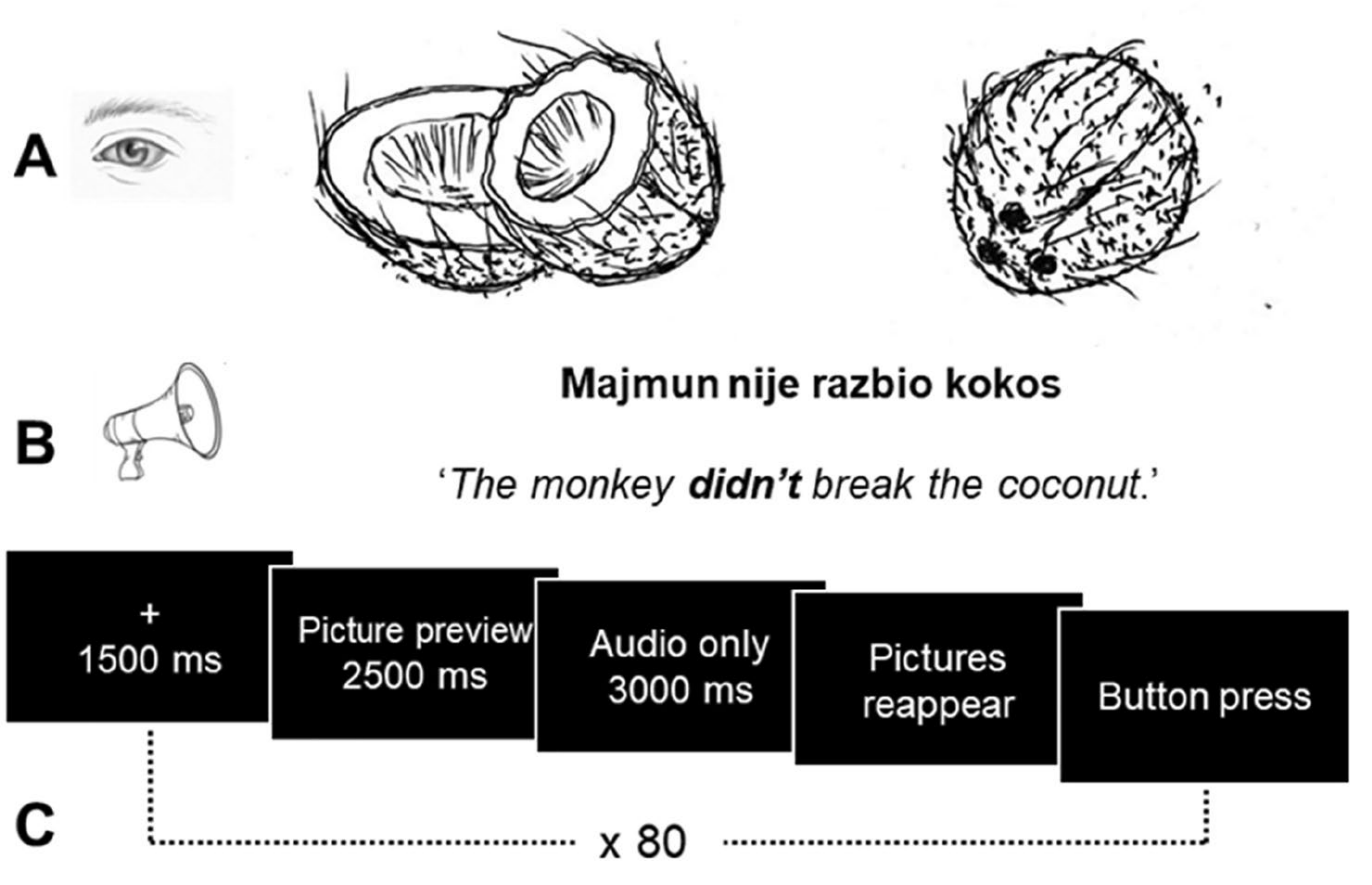
Experiment design. (**A**) Pictures pairs showing illusory vs. factual alternates. (**B**) Audio-recorded linguistic input in Croatian varying between sentential negation, negative concord, and affirmative sentences. (**C**) A trial sequence including a fixation cross, picture preview, blank screen with audio input, followed by pictures reappearing in their original positions and shown until button press.

After the instructions, the sequence for a single trial consisted of a fixation cross in the middle of the screen (1500 ms), followed by a picture preview (2500 ms), as shown in Fig. 1. After this preview, the pictures disappeared, and the participants were played the target sentence (3000 ms). Then, the pictures reappeared and were displayed until button press. While a typical blank screen paradigm^14^ only consists of two stages (visual scene first present and then absent), the current study also included a third stage (visual scene after reappearance). The rationale for adding the third stage was to have a good handle on differentiating between anticipatory and integratory processing. The second stage allowed us to zoom in on anticipatory fixations to examine early mental simulation of the factual vs. illusory, and in the third stage we focused on integratory fixations showing the proportions of looks on the factual vs. illusory after all the visual and auditory information had already been presented. The whole experiment lasted about 40 min per participant. The tasks were programmed as a web application using the jsPsych (v6.3.1) and Webgazer^40^ JavaScript libraries. All participants were tested on the same computer, keeping inter-test conditions as similar as possible. All methods were performed in accordance with the relevant guidelines and regulations.

#### Analysis

We initially present summary statistics, including the average fixation proportions for each condition and time window. We then present the results of mixed effects models, used to assess if there were significant differences in fixations launched towards representing the factual and the illusory state of affairs in the critical window during auditory input (4000–7000 ms). To do this, we constructed a series of linear mixed models using Condition (negative, nobody, positive) and Fixation target (factual, illusory) as fixed factors, and Participant and Item as random factors (lme4 package, R Studio, Version 4.1.1). The dependent variable was the total proportion of fixations, default (treatment contrast) coded. The random effect structure was kept maximal. The formula was lmer (fixtotal ~ 1 + target * condition + (1 + target * condition | participant) + (1 + target | item).

The above model analysed *whether* there were more fixations to the target in the critical window. We were also interested in *when* such a preference emerged. We thus subjected the timecourse data to a divergence point analysis^41^ between fixations on the factual vs. the illusory to estimate the onset of the experimental effect in each of the three conditions. For this analysis, fixations were grouped into bins of 200 ms. A linear model of weighted empirical logits^42,43^ was applied in each bin and the first of any three consecutive bins where looks to the factual were significantly more than to the illusory was considered the onset of the experimental effect. The data were then reshuffled within participants, conditions and timebins, and the procedure repeated 2000 times, yielding 2000 bootstrapped onsets and a 95% confidence interval using the percentile method. To determine whether onset differences were significant between two conditions, we subtracted one bootstrap distribution from the other and concluded there was evidence of a significant difference if the 95% confidence interval of the difference distribution did not contain zero^41^.

### Results

**Figure 2:**
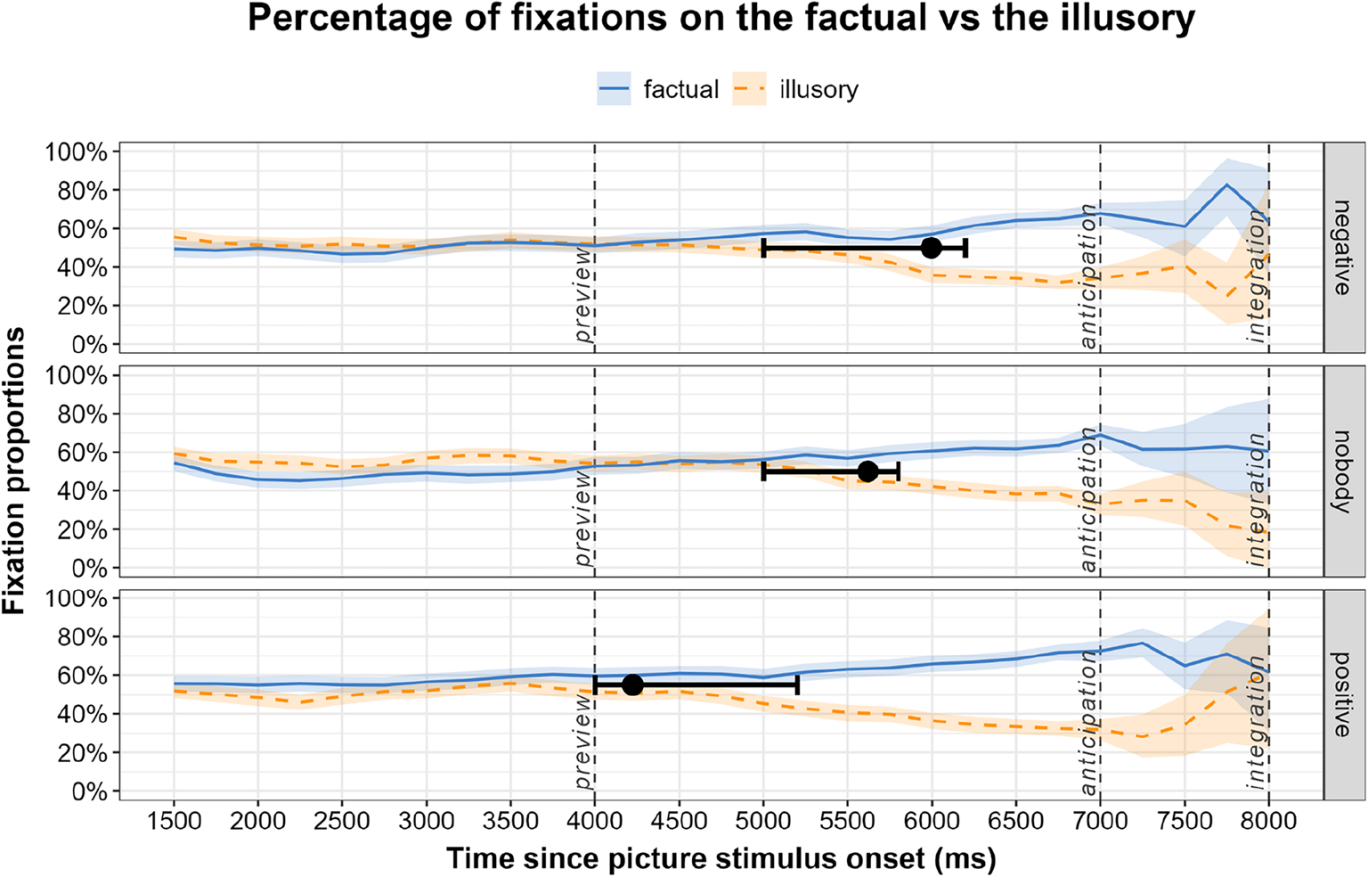
The lines show average proportions of fixations on the factual and the illusory alternates during picture preview, anticipation (audio presentation in the absence of pictures), and integration (after picture reappearance) for the Croatian group. The shading shows 95% confidence intervals. The black whiskers superimposed onto the fixation curves show divergence points and their 95% percentile confidence intervals.

Figure 2 shows the fixation proportions time-locked to the picture onset in the preview window, to the audio onset in the anticipation window, and to the picture reappearance in the integration window, calculated from correct responses only (mean proportion of correct responses for positive: 100%, SD = 0; negative 99.11, SD = 0.89; nobody: 99.11, SD = 0.88). During anticipation, the average fixation proportions on the factual were comparable in the two negation conditions (M = 58.0, SD = 46.0 for negative concord/*nobody;* M = 57.2, SD = 46.0 for sentential negation/*negative*), both exceeded by fixations on the factual in the control/*positive* condition (M = 63.4, SD = 44.5). Overall, fixations on the factual significantly exceeded fixations on the illusory, *β* = 0.21 *SE* = 0.04 *t* = − 5.05, *p* < 0.001. The effect sizes for the anticipation window, as measured by Cohen’s *d*, were *d* = 0.29 for the *negative* condition (factual: M = 57.25, SD = 45.99 vs. illusory: M = 44.04, SD = 45.82), *d* = 0.22 for the *nobody* condition (factual: M = 57.97, SD = 45.99 vs. illusory: M = 47.59, SD = 46.33), and *d* = 0.47 for the *positive* condition (factual: M = 63.40, SD = 44.53 vs. illusory: M = 42.34, SD = 45.73). This pattern of results resembled the one in the integration window, where the average fixation proportions on the factual were also comparable in the two negation conditions (M = 66.0, SD = 44.3 for *nobody*; M = 57.2, SD = 46.0 for *negative*), and the highest fixations proportion on the factual emerged in the *positive* condition (M = 72.4, SD = 41.5). During integration too, overall fixations on the factual significantly exceeded those on the illusory, *β* = 0.34 *SE* = 0.04 *t* = − 7.64, *p* < 0.001. The effect sizes for the integration window were *d* = 0.73 for the *negative* condition (factual: M = 67.13, SD = 43.73 vs. illusory: M = 35.12, SD = 44.37), *d* = 0.75 for the *nobody* condition (factual: M = 66.02, SD = 44.27 vs. illusory: M = 33.04, SD = 44.13), and *d* = 0.94 for the *positive* condition (factual: M = 72.43, SD = 41.55 vs. illusory: M = 32.14, SD = 43.94).

**Figure 3:**
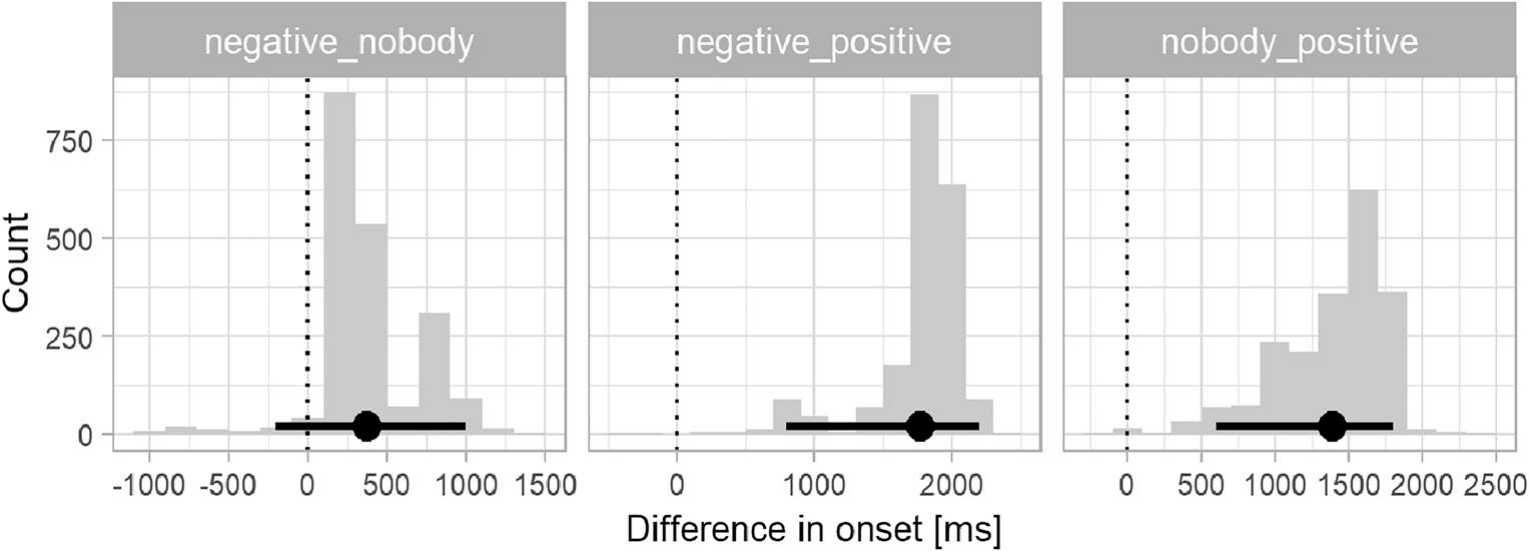
Distribution of differences in divergence points between negation conditions for the Croatian L1 group. Millisecond differences in onsets are shown on the x axis, the frequency of differences in each time bin on the y axis. Points and error bars indicate bootstrap means and 95% percentile confidence intervals. The dotted vertical line indicates a between-condition difference of zero.

Next, the mean bootstrapped divergence points and confidence intervals, superimposed on the fixation curves in Fig. 2, were visually earlier in the *nobody* condition (DP = 5620 ms after picture stimulus onset, 95% CI 5000–5800 ms) than in the *negative* condition (DP = 5996 ms, 95% CI 5000–6200 ms). As expected, the point of divergence was earliest in the control/*positive* condition (DP = 4224 ms, 95% CI 4000–5200 ms). To quantify whether the differences in onset times were significant, we generated difference distributions corresponding to our three comparisons of interest, namely *negative* vs. *nobody, negative* vs. *positive, nobody* vs. *positive.* Figure 3 shows the distribution of differences in divergence points between conditions in the Croatian group. The two histograms on the right are of interest. They suggest that both negation conditions had significantly later onsets than the *positive* condition as neither of their 95% CIs contained zero as a plausible between-condition difference: mean *negative* vs. *positive* onset 1772 ms, 95% CI 800–2200 ms; mean *nobody* vs. *positive* onset 1396 ms, 95% CI 600–1800 ms. The mean onsets did not differ significantly between the two negation conditions (left panel; 376 ms, 95% CI − 200 to 1000 ms).

We note that for the *nobody* and *negative* conditions, the divergence point analysis suggested there were two clusters of onsets at similar times in both conditions. To quantify whether either of these clusters was more prominent in one condition, we computed 95% highest density intervals (HDIs). For the *negative* condition, the HDI suggested more density in the later cluster (6000–6200 ms). For the *nobody* condition, the HDI was similar to the percentile CI reported above (5200–6000 ms). The HDIs for the negation conditions still suggested overlapping distributions, although to a lesser extent than the percentile CIs. The 95% HDI of the *positive* condition did not overlap with either of the negation conditions (4000–5000 ms). Since the nature of the two clusters in the negation conditions was unknown, we assumed that they belonged to one process driving preferential looks to the target. Based on this assumption, in Fig. 2 we present the mean onset estimate—which is naturally biased toward the highest density of the distribution—and its 95% percentile confidence interval in order to take both clusters into account.

### Discussion

Preferential eye movements towards the position of the factual state of affairs launched soon after Croatian listeners heard negation are interpreted as evidence of mental simulation of the factual during negation processing. These findings extend previous observations^14^ of language-mediated eye movements in the absence of a visual world triggered by verb semantics (e.g., hearing *to read* or *to eat* generated anticipatory looks where readable and edible objects respectively) to the domain of negation processing. Crucially, this domain boasts variation not only across but also within languages, which served here as a springboard for testing hypotheses about the predictive power of different negation types. The key variation within Croatian^24^ lies between negative concord, which provides a double cue, and sentential negation, which provides a single cue. Points of divergence in fixations on the factual vs the illusory emerged earlier for the double cue condition than for the single cue condition. However, the mean divergence point onsets did not differ significantly in the two negation conditions, so this finding from the blank-screen paradigm cannot provide support for the hypothesis that, in Croatian, the power to predict the factual state of affairs changes as a function of negation type. In the context of the number of cues available for predictive processing, negative concord with a double cue does not appear to substantially boost predictive power compared to sentential negation with a single cue.

In terms of processing steps, more robust fixations on the factual from early on in the trajectories of both negation conditions are taken as manifestations that Croatian listeners process the factual state of affairs directly^31,34^, without the need to detour through the illusory. This finding aligns with the view that negation may be represented symbolically rather than iconically^32,33^, possibly as a mental tag^35^. Our fixation trajectories are inconsistent with the two-step processing model advocated by^4^ as fixations on the factual prevail those on the illusory from the earliest points of divergence. Listeners continued to fixate on the factual more than on the illusory in the integration time window until they were done with processing, suggesting that negation unambiguously cued the factual referent throughout prediction and integration. It is the anticipatory looks that Croatian native listeners directed towards pictures with the factual state of affairs that demonstrate the presence of probabilistic computations that include the visual features of objects^12,13^. This finding aligns with^44^, who showed that comprehenders launch anticipatory looks towards objects based on placement verbs before they hear the noun, and also with^45^, who showed that listeners activate the shape characteristics of the target object before the noun is heard.

These findings show that negation serves as a predictive cue in Croatian sentence processing. The differences found in the proportions of anticipatory looks helped to establish that the degree of prediction may vary in accordance with the number of cues that a specific type of negation provides, but this variation was not substantially different between negative concord and sentential negation in Croatian. In the next step, we examine the extent of negation-mediated predictive behaviour in English native listeners.

## Experiment 2: native English listeners

### Method

#### Participants

Forty-two native English speakers (mean age 25.3 years, range 19–34, 6 males) took part in the experiment. They were recruited at the University of Auckland and the University of Melbourne. Each participant received a gift voucher for participation. The inclusion criteria were fluency only in English, have normal or corrected-to-normal vision and no language impairments.

#### Materials, procedure and analysis

The experimental design and all procedural steps mirrored those described in Experiment 1, except for the linguistic input. The Croatian sentences were translated into English, and the translations were independently checked for accuracy by two Croatian-English bilinguals. The English sentences were read out and recorded by a female native English speaker. The 84 English audios also included 20 × sentential negation (e.g., *The monkey didn*’*t break the coconut*), 20 × negative quantifier negation (e.g., *Nobody broke the coconut*), 20 × affirmative control sentences (e.g., *The monkey broke the coconut*), 20 × filler sentences (e.g., *The fish got burnt*’), and 4 × training sentences (e.g., *The sailor tied the knot*). The presentation length of the English audio stimuli was kept constant (2500 ms). The analytical procedures were identical with those used in Experiment 1.

### Results

**Figure 4:**
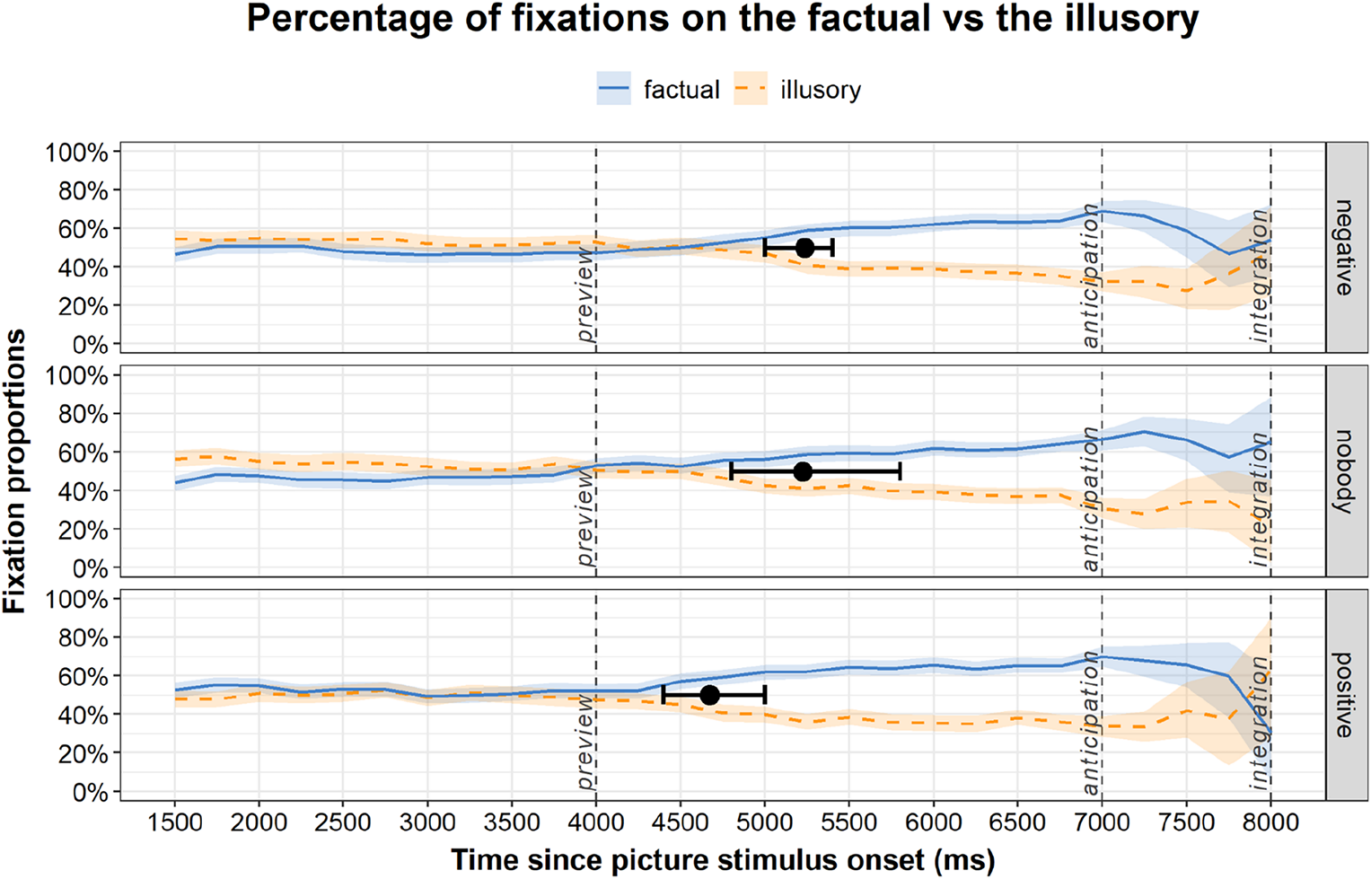
The lines show average proportions of fixations on the factual and the illusory alternates during picture preview, anticipation (audio presentation in the absence of pictures), and integration (after picture reappearance) for the English L1 group. The shading shows 95% confidence intervals. The black whiskers superimposed onto the fixation curves show divergence points and their 95% percentile confidence intervals.

In Fig. 4, average fixation proportions of the English listeners are shown during preview, anticipation, and integration, calculated from correct responses only (mean proportion of correct responses for positive: 99.56%, SD = 1.79; negative 98.11, SD = 3.07; nobody: 98.56, SD = 2.53). In the anticipation window, the average fixation proportions on the factual were comparable for the two negation conditions (M = 58.1, SD = 44.7 for negative quantifier negation/*nobody;* M = 57.2, SD = 45.2 for sentential negation/*negative*), both exceeded by fixations on the factual in the control/*positive* condition (M = 61.1, SD = 44.5). Across conditions, fixations on the factual significantly exceeded fixations on the illusory, *β* = − 0.17 *SE* = 0.04 *t* = − 4.10, *p* < 0.001. The effect sizes for the anticipation window, as measured by Cohen’s *d*, were *d* = 0.31 for the *negative* condition (factual: M = 57.21, SD = 45.22 vs. illusory: M = 43.01, SD = 45.12), *d* = 0.34 for the *nobody* condition (factual: M = 58.14, SD = 44.74 vs. illusory: M = 42.94, SD = 44.78), and *d* = 0.48 for the *positive* condition (factual: M = 61.06, SD = 44.47 vs. illusory: M = 39.56, SD = 44.44). A similar pattern of results was found in the integration window, where the average fixation proportions on the factual in the two negation conditions were alike (M = 66.8, SD = 43.6 for *nobody*; M = 65.9, SD = 44.1 for *negative*). The fixation proportion on the factual in the *positive* condition was 68.5% (SD = 42.7). In the integration window too, the overall fixations on the factual significantly exceeded those on the illusory, *β* = − 0.39 *SE* = 0.05 *t* = − 7.29, *p* < 0.001. The effect sizes for the integration window were *d* = 0.78 for the *negative* condition (factual: M = 65.94, SD = 44.10 vs. illusory: M = 31.97, SD = 42.67), *d* = 0.84 for the *nobody* condition (factual: M = 66.78, SD = 43.64 vs. illusory: M = 30.71, SD = 41.76), and *d* = 0.79 for the *positive* condition (factual: M = 68.50, SD = 42.67 vs. illusory: M = 34.41, SD = 43.25).

In the next step, we calculated the bootstrapped divergence points for the three conditions, and we added the bootstrap confidence intervals onto the fixation curves in Fig. 4. The results based on 2000 bootstrap replicates showed that, different from the Croatian group, in the English group the fixations diverged at similar points in the *nobody* condition (DP = 5224 ms, 95% CI 4800–5800 ms) and in the *negative* condition (DP = 5236 ms, 95% CI 5000–5400 ms). The fixation curves diverged earliest in the *positive* condition (DP = 4672 ms, 95% CI 4400–5000 ms).

**Figure 5:**
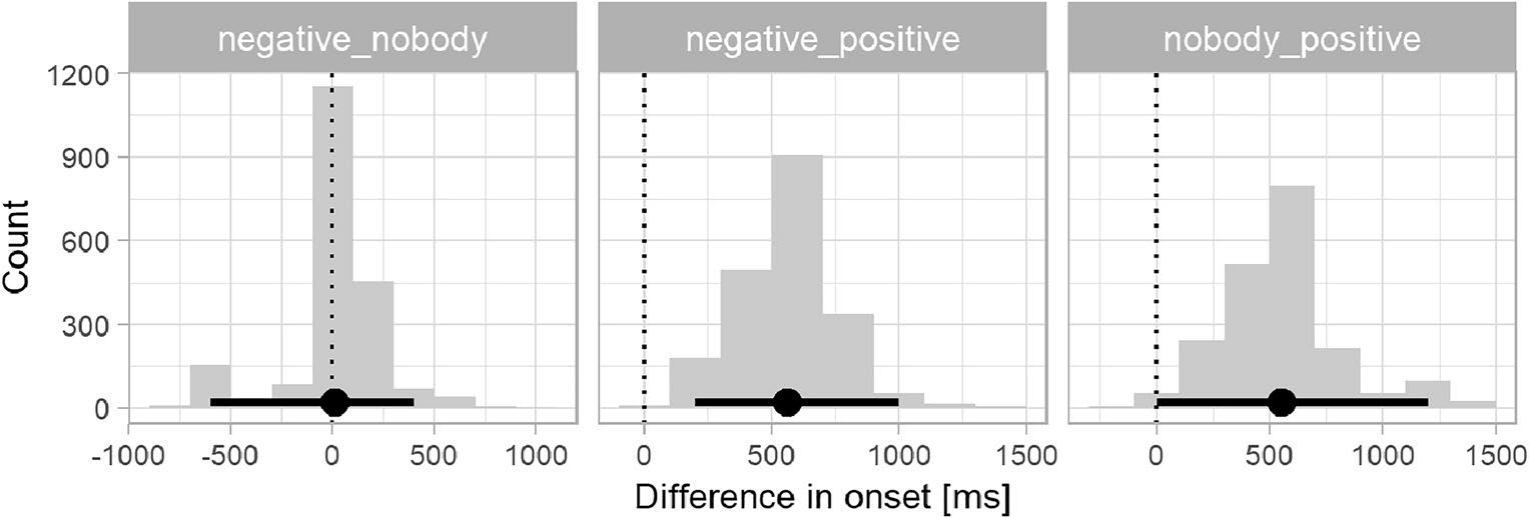
Distribution of differences in divergence points between negation conditions for the English group. Ms differences in DP onsets are shown on the x axis, the frequency of differences in each time bin on the y axis. Points and error bars indicate bootstrap means and 95% percentile confidence intervals. The dotted vertical line indicates a between-condition difference of zero.

Figure 5 shows the distribution of differences in divergence points between conditions in the English group. The two histograms on the right suggest that, similar to the Croatian group in Expt. 1, the onset difference between sentential negation and affirmatives/*negative_positive* (M = 564 ms, 95% CI 200–1000 ms) was significant. The lower bound of 95% CI of the negative quantifier and affirmative, i.e., the *nobody_positive* contrast was zero and thus was not statistically significant (M = 552 ms, 95% CI 0–1200 ms), but the overall difference distribution did suggest a between-condition difference similar to the Croatian group. The onset difference between sentential negation and the negative quantifier was consistent with zero (M = 12 ms, 95% CI − 600 to 400 ms). Based on observation rather than statistical tests, the onset differences between negation types do not support the predicted pattern that a considerably smaller between-negation onset difference would emerge in the English group than in the Croatian group.

### Discussion

The results from Experiment 2 show that native English listeners too launch eye movements towards the factual more than to the illusory soon after hearing negation. This finding from anticipatory fixations on a blank screen provides evidence that English listeners also mentally simulate the factual state of affairs during negation processing. The key variation within English is between the negative quantifier, which was used to provide a single cue in a sentence-initial position, and sentential negation, which also provided a single cue but later on in the sentence in a preverbal position. Our hypothesis was that the predictive power of different negation types should be less different within English (single vs. single cue) than within Croatian (double vs. single cue). English listeners’ fixations on the factual diverged from those on the illusory at similar points in the acoustic life of negation via a negative quantifier and sentential negation. Our interpretation is that linguistic mediation of English listener’s eye-movements in the absence of a visual world was equally strong in the negative quantifier negation condition as in sentential negation. The results from divergence point onsets did not support the hypothesis that having two cues vs. one (Croatian) would come with more robust difference in the predictive power than having two different single cues (English). Other than the number of cues, or their sentential position, the power to predict the factual state of affairs in response to hearing English negative sentences could also have changed as a function of directness in negative polarity marking. Sentential negation with *didn’t* as a separate, more direct marker of negative polarity of the verb could have facilitated correct predictions faster than a less direct negation via a negative quantifier. Mental simulation of the factual, when cued by sentential negation, may have required less processing effort (similarly to the binary condition in^33^) compared to the negative quantifier that specifies the context arguably less. However, anticipatory fixations in the two negation types in English varied little. We return to this point in the general discussion.

As shown in the trajectories of Fig. 4, more fixations on the factual than on the illusory soon after encountering negation suggest that English listeners process the negative/factual directly^31,34^ rather than simulating the positive/illusory first. This finding, comparable with that from Croatian listeners, aligns more closely with the symbolic/one-step^32,33^ rather than with the iconic/two-step processing model^4^. Fixations on the factual exceeding those on the illusory from the onset of divergence during anticipation, and at no point falling under the average fixations on the illusory until the end of processing in the integration window, indicate that listeners perform probabilistic computations in their mental simulations that rely more on visual features of the factual than the illusory.

In sum, these findings from Experiment 2 show that native English listeners as well make use of negation to anticipate the factual state of affairs in sentence processing. Negligible differences in the proportions of anticipatory looks, not varying as a function of negation type, bring new insights that, in English, the onset of prediction does not depend on the polarity of the verbal cue that characterises a specific type of negation.

## General discussion

This study breaks new ground in examining the effects of crosslinguistic variation in negation on predictive processing. Soon after they heard the verb being mentioned, participants mentally simulated the factual state of affairs by fixating on where the corresponding picture had been positioned. While other studies found verbal semantics to serve as a predictive cue to signal the target object^12^ or its positional characteristics^44^, this study extended the scope of inquiry to the predictive power of negative verb phrases. Adding a crosslinguistic level helped to show how two sets of different markings of negation, in Croatian and in English, modulate predictive sentence processing. Sets of data from both native listener groups indicated that looks launched towards the location of the appropriate/factual exceeded looks towards the inappropriate/illusory state of affairs shortly after the verb phrase. Appropriate looks varying according to the type of linguistic input (negative vs affirmative sentences) show that listeners are able to dynamically modify representations of what is factual and what is illusory in a given context.

What caused the eyes to fixate somewhere specific when the screen was blank? Previous research showed that eye movements triggered in the course of processing linguistic input do not depend on a co-presentation of a visual scene with verbal material^14^. Even when the factual was not actual, i.e., no scene was concurrently presented during linguistic input, eyes moved to the location of the appropriate referent. This behaviour presents evidence from the domain of negation processing that the informational basis of eye fixations may not necessarily lie in the actual location of the referent. Instead, it seems that eye movements can be triggered by the location of that referent as it was mentally represented when the actual scene was absent. A possible mechanism is proposed by^46^. They suggest that spatial coordinates are integral to the memory trace associated with each visually inspected object as part of a scene, and activation of that trace linguistically co-activates the experience of seeing the location of that object, which automatically triggers eye movements towards the relevant location. Within this proposal, the memory of a scene showing the factual, including spatial coordinates relative to the scene showing the illusory, initiated and navigated eye movements through linguistic input. The level of detail necessary to extract from a visual scene to form a representation is a resonant topic more generally^47^. Within negation research, it remains to be tested in future designs, e.g., through variations in picture preview, what constitutes sufficiently informative visual input to support differential representations of the factual vs. the illusory. Other than variations in picture preview, a further potential enhancement of the design could be to vary agency in the sentences. This is because a sentence with an agent such as *The monkey didn’t break the coconut* does not exclude the possibility that some other agent might have broken the coconut. Ambiguous sentences could have delayed fixations on the factual due to added complexity. Alternatively, ambiguous sentences could have driven early commitments to the most plausible target picture so that more cognitive resources could become available to process the fast stream of auditory input. While the effect of presence/absence of an agent was beyond the scope of this study, future studies might consider manipulating this aspect explicitly or balancing the materials to address the potential confound. A critical reader may also wonder about the extent to which participants’ anticipatory eye movements could have been influenced by the unusual use of the blank-screen paradigm in this study (i.e., including picture reappearance). Participants knew that the pictures would reappear, which may have impacted how and when they launched eye movements. There are a few studies showing that fixation patterns when participants are not asked to perform an explicit task (as in^12,14,48^) replicate results from a design with an explicit task (involving visual scene memorisation, as in^49^). Still, the specific epistemological implications of a two-stage blank-screen paradigm and its modified version when the pictures reappear (*i.e.*, whether these different kinds of blank-screen tasks are hospitable to comparable degrees of anticipatory looks) are yet to be identified.

Possibly the most consistent set of findings is that participants tended to initially focus their attention on the picture representing the factual rather than the illusory state of affairs. This was the case regardless of negation type and language group. Could it be that the chosen experimental setup activated mental representations of the factual state even before anticipatory eye movements became relevant? In other words, could the results be influenced by practice effects since participants were exposed to the same pictures paired with the same verbs three times throughout the experiment? There are two reliable signals that strong training effects were unlikely. First, the experimental design provided a substantial picture preview time of 2500 ms. If the correct picture had been predictable even before linguistic input, for instance via remembering previous picture pairs, one would expect pronounced divergences in fixations already towards the end of the picture preview. This was not the case in any of the conditions in either language. Second, the target picture differed across conditions (e.g., the factual was represented by the whole coconut in the negative conditions but by the broken coconut in the positive condition), which lessened picture predictability in the pre-verbal time window. While some training effects could have emerged as the experiment unfolded, in terms of validity it was reassuring to observe that most divergence points occurred between the verb and the referential expression (e.g., *the coconut*). To further strengthen the design, future versions of the experiment could limit picture pair presentation to just a single exposure for each participant, vary event types/verbs per picture pair, or combine unrelated pictures out of which only one meets the selection restrictions of the verb.

Divergence in fixations observed during auditory input implies linguistic modulations of eye movements. Tighter control of linguistic input could be achieved by presenting the content verb at exactly the same point in the input stream, both in sentential negation (*Sara didn’t pierce*) and no later in negative quantifier negation (*Nobody pierced*). This modification could be complemented with a short pause after the verb to increase the potential for launching fixations before the referent gets mentioned^14^. The current methodology did not compromise ecological validity by breaking the speech flown with potentially unnatural pauses, still, the majority of fixations diverged shortly after verb mention. One exception was the positive condition in Croatian, where fixations seem to have diverged earlier than at verb offset. A tempting explanation is that participants may have noticed that each picture pair was presented twice with negation and once with affirmation, picking up a pattern as the experiment unfolded. There are two points that strengthen the opposite, namely that trial order randomisation minimised target picture predictability, and that preverbal divergence points were rare for the same condition in the English group, suggesting that most DPs were language-modulated. Unwanted predictability of what may turn out to be the factual state could be further reduced by using a four-picture display as is common in the visual world paradigm^12,13^ co-presenting the factual target, the illusory competitor, and two distractors that do not meet the verb’s selection restrictions. Such an extension would be straightforward to employ, yet caution is needed with direct imports of design features from the visual world into the blank screen paradigm because perceptually dominant or otherwise imbalanced distractors could easily swallow anticipatory looks that are rare on a blank screen anyway. A principled way forward could be to select pictures based on norms for perceptual and action strength^50^. Alternatively, future studies could find it advantageous to add a pre-screen task in which participants would need to recall pictures from pairs or quadruplets after preview. Then, recall accuracy could be added as a fixed factor into models to wash out potential recall-related variation.

Moment-by-moment coordination of mental states examined through eye-fixation data indicates that an event that *isn’t* can be as salient as a concrete observed event. More saccades towards the factual negative state throughout the anticipatory and integratory windows across negation conditions and languages raise challenges for the two-step model of negation processing as advocated by^6,51^ and^4,5^. Instead, we interpret the results observed during the anticipation window as evidence that different forms of negation act like a strong compass that from early on navigates comprehenders to mentally simulate the negative state directly, as suggested for instance by^34^, rather than via a positive state. Early fixations towards the factual signal that negation constitutively structures mental simulations about what *is not*. By effectively cueing absences or negative states in a fast matching of speech with visual input, the role of negation in sensory simulations appears to be at least on some level symbolic rather than purely iconic^32,33^. Direct simulation of the negative state instead of managing an additional affirmative state can be attributed to working memory constraints, as a single representation is cognitively less taxing to keep in mind. Even though anticipatory eye-movements emerged faster in affirmation than in negation for both language groups, dominance of fixations on the factual throughout the processing stream strongly suggests one longer step for negation, giving little ground to assume an extra second step through the positive.

Another important new insight from this study is the absence of a robust variation in the strength with which structural cues in English and Croatian provide information about how factual states are understood. Analyses of fixation trajectories showed that divergence points were similar across negation conditions within English (negative quantifier vs. sentential negation, i.e., single vs. single cue) and also within Croatian (negative concord vs. sentential negation, i.e., no significantly earlier divergence with the double cue than with the single cue). Negative concord cannot thus be viewed as a more heavy-weight top-down signal that builds a faster direct connection with the negated meaning in a symbolic format than sentential negation does. Findings from anticipatory fixation analyses with Croatian and English participants bring a finer level of grain to the extant accounts of negation processing in one single step^33^. Future tests could tell whether similar effects of different negation types extend beyond anticipatory processing, for instance via methodological triangulation using a speeded sentence-picture verification task^6,51^ combined with recall, or an EEG study testing brain signatures during response inhibition^52^ across negation types. Also, a future study using a fully crossed design with four groups, namely English users of Croatian tested in both English and Croatian as well as Croatian users of English tested in both Croatian and English, would provide the optimal testbed necessary for direct crosslinguistic comparisons statistically rather than based on pattern observation.

Using a crosslinguistic comparison of processing negative and affirmative sentences by native listeners of Croatian and English brought new insights into the predictive mechanisms that negation triggers across and within languages. Perhaps the most intriguing new contribution is the time-course of mental simulations revealed when the visual world is a mere memory. Launching eye movements towards the location of the factual underlines the hypothesis that mental simulation of the factual negative state of affairs is automatic and processed directly rather than via the illusory positive.

## Data Availability

The full datasets, analysis codes, eye-fixation visualisations per group, by participant as well as by item, all picture stimuli and auditory stimuli for the present study are available through the Open Science Framework at https://osf.io/czyx7/.
